# Left Ventricular Non-compaction Cardiomyopathy: The Key to Its Diagnosis and Implications for Management

**DOI:** 10.7759/cureus.47121

**Published:** 2023-10-16

**Authors:** Angela E Gallucci, Meghan R Grewal, Brooke T Alexander, Arianna M Heyer, Yvonne M Diaz

**Affiliations:** 1 Internal Medicine, University of Miami Miller School of Medicine, Miami, USA; 2 Urology, University of Miami Miller School of Medicine, Miami, USA; 3 Internal Medicine, University of Miami Miller School of Medicine, Jackson Memorial Hospital, Miami, USA

**Keywords:** thromboembolism, anticoagulation, left ventricular dysfunction, heart failure, ventricular non-compaction, trabeculations, non-compaction cardiomyopathy

## Abstract

Left ventricular non-compaction (LVNC) cardiomyopathy is a condition with increasing prevalence as cardiac imaging technology improves, although there is currently no diagnostic gold standard. Characterized by the presence of a bilayered myocardium with prominent trabeculations, LVNC cardiomyopathy has a wide range of presentations, from asymptomatic to severe heart failure, thromboembolism, and sudden cardiac death. We present the case of a 62-year-old male who was admitted for a heart failure exacerbation with a worsening ejection fraction and signs of increased trabeculations of the left ventricle on an echocardiogram. We highlight the rarity of this condition, especially when diagnosed via echocardiogram, and the importance of considering anticoagulation as part of the treatment plan.

## Introduction

Left ventricular non-compaction (LVNC) cardiomyopathy is a rare condition characterized by the presence of a bilayered myocardium with prominent trabeculations. The prevalence of LVNC cardiomyopathy has increased with advancements in cardiac imaging technology, although there is currently no diagnostic gold standard [1]. In one systematic review of the prevalence of LVNC cardiomyopathy in adults, estimates for LVNC were consistently higher amongst cohorts diagnosed via cardiovascular magnetic resonance (CMR) imaging (14.79%) compared with echocardiograms (1.28%) [2-4]. Left ventricular non-compaction cardiomyopathy has a wide range of presentations, from asymptomatic to severe heart failure, thromboembolism, and sudden cardiac death. Once the diagnosis is made, consideration for genetic testing and the need for anticoagulation is paramount, as these can have significant prognostic implications [5]. We report the case of a 62-year-old male who presented with uncontrolled hypertension and frequent heart failure exacerbations and was found to have signs of LVNC on an echocardiogram. This article was previously presented as a meeting abstract at the 2023 Florida Medical Association Meeting on July 29, 2023.

## Case presentation

A 62-year-old male with a history of hypertension, hyperlipidemia, and heart failure with reduced ejection fraction(HFrEF) (ejection fraction (EF) at 15% to 20%) presented with two days of bilateral lower extremity swelling and shortness of breath. His vital signs on this admission were remarkable for a respiratory rate of 30, blood pressure of 143/110, and oxygen saturation of 95% on room air. On a physical exam, the patient had diffuse crackles bilaterally with inspiratory wheezes and grade 3 bilateral lower extremity edema. His labs revealed an elevated brain natriuretic peptide (BNP) at 9000, and imaging showed diffuse interstitial edema, consistent with a heart failure exacerbation. The patient initially required bilevel positive airway pressure (BiPAP) for increased breathing and IV diuresis for three days.

**Figure 1:**
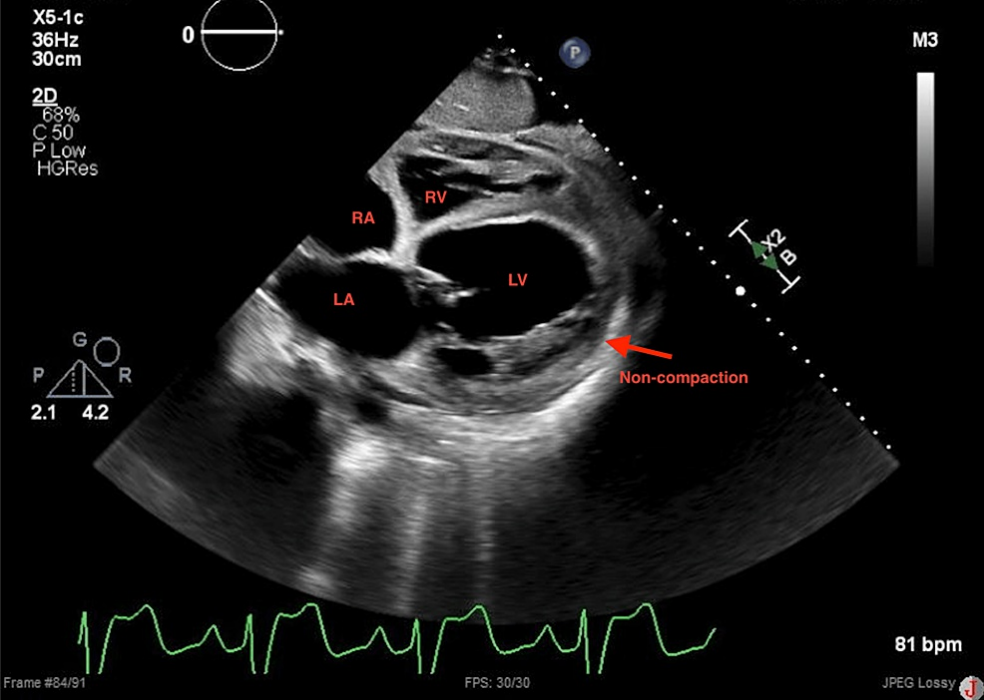
2D transthoracic echocardiogram (subcostal view) showing a dilated left ventricle and signs of LVNC LVNC: Left ventricular non-compaction

**Figure 2:**
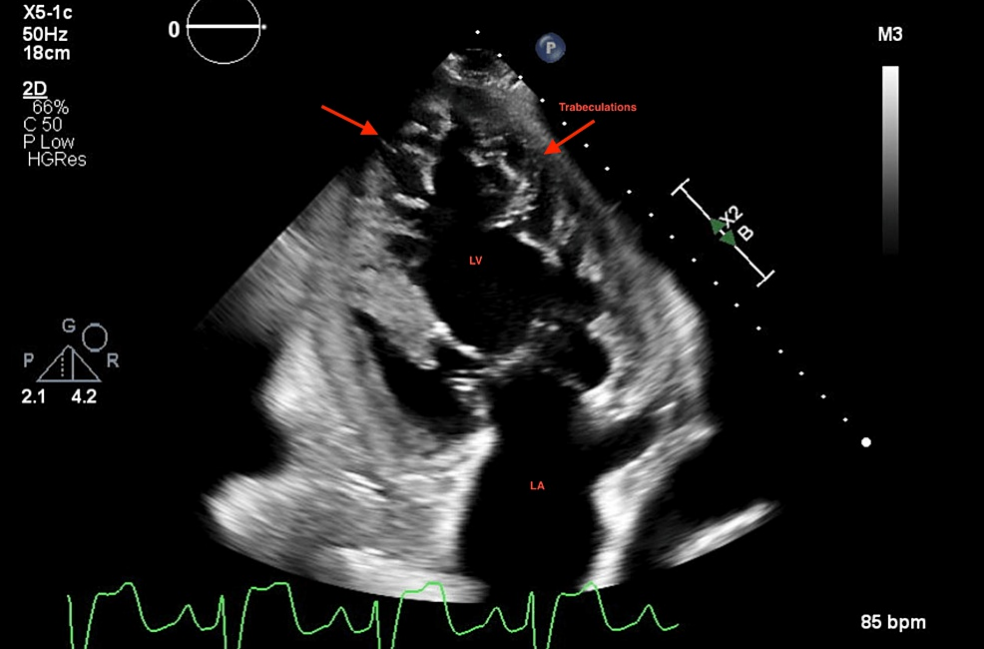
2D transthoracic echocardiogram (two-chamber view) of the patient with pronounced trabeculations of the left ventricle (red arrows)

**Figure 3:**
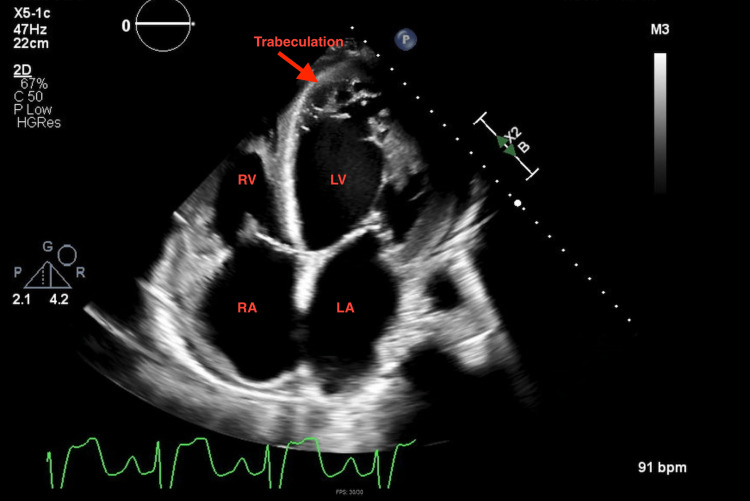
2D transthoracic echocardiogram (four-chamber view) showing a region of trabeculation at the left ventricular apex

A repeat echocardiogram at this admission showed a worsening EF of 10% to 15% with a severely dilated left ventricle (LV), grade 2 diastolic dysfunction, and severe mitral regurgitation. Also noted was the presence of increased trabeculation at the left ventricular apex, concerning for non-compaction cardiomyopathy (Figures 1-3).

**Figure 4:**
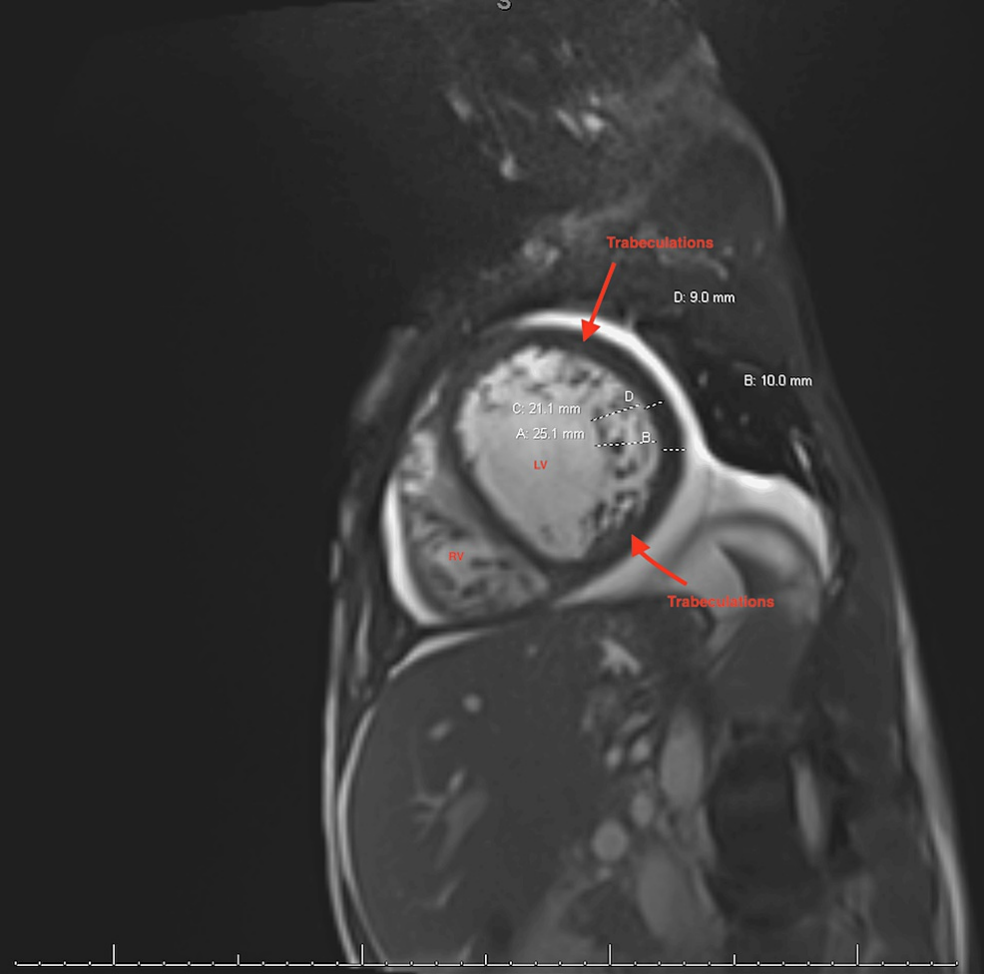
Cardiac MRI (sagittal view) with hypertrabeculations meeting the criteria for LVNC cardiomyopathy LVNC: Left ventricular non-compaction

The patient responded well to treatment and was successfully weaned to room-air oxygen. Clinical resolution of his heart failure exacerbation was evidenced by improvement in his shortness of breath, bilateral lower extremity edema, and net negative fluid balance. He was placed on telemetry for monitoring at the time of admission, which was remarkable for sinus tachycardia with occasional multifocal premature ventricular contractions (PVCs). A cardiac MRI was performed for diagnostic completion and confirmed hypertrabeculation that meets the criteria for LVNC cardiomyopathy based on the Jenni criteria with a ratio of noncompacted diastolic myocardium to compacted myocardium of 2.5 (Figure 4).

**Figure 5:**
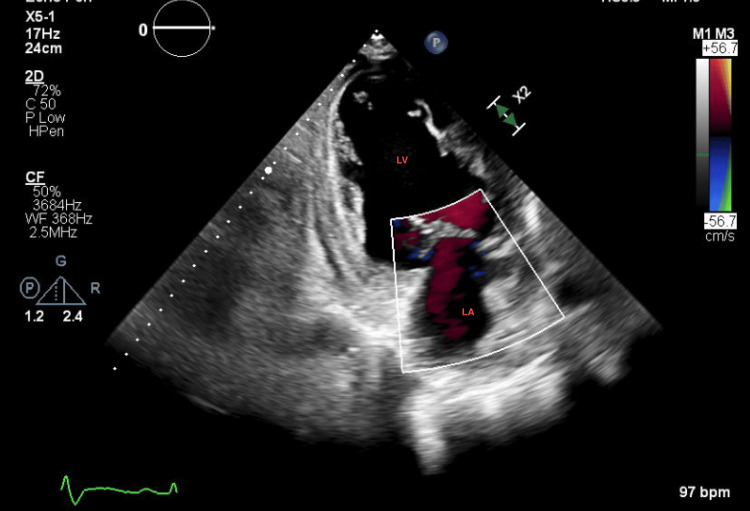
2D transthoracic echocardiogram (two-chamber view) of the patient in March 2021 that did not report trabeculations of the left ventricle

Upon careful chart review, the patient had two similar admissions over the last several months. Due to financial constraints, he was unable to follow up in the cardiology clinic. The diagnosis of LVNC cardiomyopathy was first mentioned on an echocardiogram in 2011. Repeated echocardiograms since then have been inconsistent in reporting LVNC (Figure 5).

The patient was initially started on apixaban for anticoagulation about five months prior; however, this medication and the diagnosis of LVNC were often absent from the electronic medical record. Despite many heart failure exacerbations over the last year, until this admission, the patient was unaware of his specific diagnosis and the possible need for anticoagulation or the risk of requiring an implantable cardioverter-defibrillator (ICD). We also discussed the option of genetic testing, which he declined at the time. The patient denied any known family history of LVNC cardiomyopathy. At the time of discharge, the patient was prescribed apixaban 5 mg twice daily, sacubitril/valsartan 24 to 26 mg twice daily, aspirin 81 mg daily, carvedilol 3.25 mg twice daily, furosemide 40 mg daily, dapagliflozin 10 mg daily, and rosuvastatin 20 mg daily.

## Discussion

Left ventricular non-compaction cardiomyopathy is a rare condition that is characterized by the appearance of prominent trabeculation of the LV with deep intertrabecular recesses [2]. These intertrabecular recesses are continuous in the LV cavity and filled with blood but remain separate from the epicardial coronary arteries [2]. Left ventricular non-compaction cardiomyopathy is classified as a primary cardiomyopathy, meaning it is not caused by other underlying heart conditions such as hypertension or coronary artery disease [2,3]. The true prevalence of LVNC is not known, and there is currently no diagnostic gold standard [3,4]. Advancements in cardiac imaging technology, such as CMR, have contributed to an increase in the prevalence of LVNC cardiomyopathy. In one systematic review, the prevalence of LVNC in adults was estimated to be consistently higher among cohorts diagnosed via CMR imaging (14.79%) compared with echocardiograms (1.28%) [4].

Left ventricular non-compaction cardiomyopathy has a wide range of symptoms and complications. The condition can range from asymptomatic to severe heart failure, thromboembolism, and sudden cardiac death, which can make management extremely challenging [2]. The most common clinical presentations at the time of diagnosis are heart failure, thromboembolic events, and arrhythmias [2,3,6-9]. A variety of mutations have been reported in LVNC cardiomyopathy, the most common involving sarcomere proteins [3]. Such mutations can be sporadic or familial, with an autosomal dominant inheritance pattern reported to be the most common [3,7]. Genetic testing may therefore play an important role in diagnosis and management. One study by van Waning et al. reported a higher risk of reduced LV systolic dysfunction in patients with certain genetic mutations, with the greatest risk observed in patients with multiple mutations. There was also a high risk of adverse cardiac events in children and adults related to LV systolic dysfunction in mutation carriers, but not in sporadic cases [3]. This consideration was particularly important for our patient, who presented with a significantly reduced EF in the setting of numerous heart failure exacerbations. 

Management of LVNC cardiomyopathy involves a multidisciplinary approach, including cardiologists, geneticists, and other specialists. Treatment aims to alleviate symptoms, improve cardiac efficiency, and reduce the risk of adverse events through the use of guideline-directed medical therapy, diuresis, and blood pressure control [8]. Anticoagulation for qualified patients or those at risk for thromboembolic events is important to consider as well. It can be challenging to determine which patients qualify for anticoagulation therapy considering the low disease prevalence and the limited number of large, randomized controlled trials. A recent literature review of 174 articles by Chimenti et al. sought to address this knowledge gap. Specifically, for patients diagnosed with LVNC cardiomyopathy and associated LV dysfunction (EF <50%) without atrial fibrillation, they recommend initiating anticoagulation therapy with or without LV thrombus [10,11]. This is a class IIb recommendation and a reasonable option, according to the 2019 consensus statement by the Heart Rhythm Society [12]. The LV dysfunction places patients with LVNC at an increased risk of developing a thrombus due to blood stagnation and local myocardial injury within the deep intertrabecular recesses [11]. At-risk patients must be properly treated because the presence of an LV thrombus is associated with high rates of systemic embolism, morbidity, and mortality [11]. As in the case of our patient without a known LV thrombus or atrial fibrillation but with significant LV dysfunction, we chose to initiate anticoagulation therapy with apixaban for preventative measures. Long-term anticoagulation with direct oral anticoagulants (DOACs) is preferred in patients diagnosed with LVNC cardiomyopathy and LV dysfunction alone [11,12]. 

In the setting of severe systolic dysfunction, management with an ICD can be considered as well. There are currently no special indications for ICD implantation in patients with LVNC cardiomyopathy; rather, the decision should be made depending on the patient's clinical presentation. An ICD implantation should be considered in patients with life-threatening arrhythmias and/or a family history of sudden cardiac death [2,8]. The most common arrhythmias associated with LVNC are atrial fibrillation and supraventricular arrhythmias [2]. In patients with LVNC who appropriately receive ICD placement, there are reports of improved prognosis in addition to the prevention of cardiac arrest [2]. Cardiac risk factors such as arrhythmias and ventricular dysfunction are strongly associated with increased mortality in patients with LVNC cardiomyopathy [6]. Thus, close monitoring of patients is extremely important. Follow-up may consist of arrhythmia screenings, including electrophysiology (EP) evaluation [6]. Our patient is scheduled to visit the EP clinic due to his frequent and severe heart failure exacerbations and worsening ejection fraction.

## Conclusions

Due to a lack of a clear diagnosis for most of his adult life, our patient suffered frequent heart failure exacerbations and recurrent hospital admissions. Thus, we would like to emphasize the importance of investigating cardiac imaging findings to make an official diagnosis when the signs of LVNC cardiomyopathy are present. We recommend genetic testing as an additional diagnostic tool for high-risk patients. Though our patient declined genetic testing, we would like to emphasize the clinical role this may have for patient management. As discussed above, distinguishing genetic from nongenetic causes of LVNC can aid in predicting disease outcomes and thus have implications for disease management. Though not necessary for diagnosis, genetic testing may play a vital role in clinical care, as patients with multiple mutations are at greater risk for left ventricular systolic dysfunction and adverse cardiac events. An appropriate estimate of disease severity has important implications regarding the extent of management a clinician should consider as well, such as frequency of follow-up and the need for anticoagulation or an ICD.
